# Supplementary material for: A human cytomegalovirus prefusion-like glycoprotein B subunit vaccine elicits humoral immunity similar to that of postfusion gB in mice
Source: J Virol. 2025 May 8;99(6):e02178-24. doi: 10.1128/jvi.02178-24 (PMC12172439; doi:10.1128/jvi.02178-24)
Supplement: Supplemental material — Figures S1 to S9; Table S1. [file jvi.02178-24-s0001.docx]

**Supplementary Figures**

**
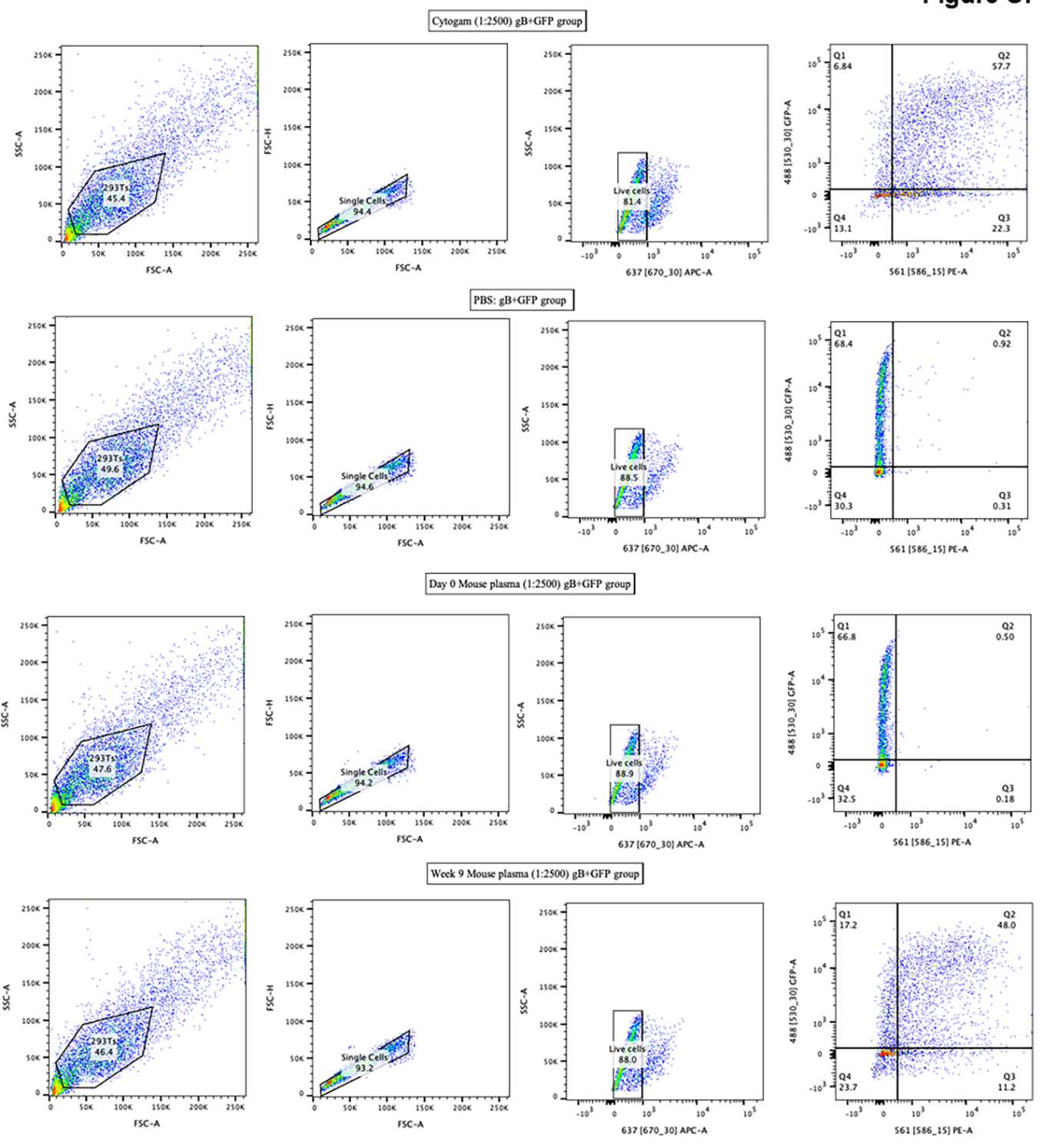
Figure S1: Flow cytometry gating strategy for measuring IgG binding to cell-associated gB using a transfected cell binding assay.** IgG binding to cell-associated gB (Towne) was measured by co-transfecting 293T cells with plasmids expressing HCMV-Towne gB and GFP, followed by incubation with mouse plasma. Cells were stained with Live/Dead Fixable Near-IR Dead Cell Stain, followed by phycoerythrin (PE) - conjugated goat-anti-mouse IgG Fc staining, and fixed with 10% formalin prior to acquisition via high throughput sampler (HTS) on the flow cytometer (Fortessa; BD). The frequency of PE+ cells was reported for each sample based on the live, singlet, GFP+ population. The average + 3 standard deviation (SD) of baseline (Week 0) samples was used as the positivity cutoff. The figure shows gating strategy for a positive control Cytogam, PBS, and mouse plasma at Day 0 (background) and Week 9.

**
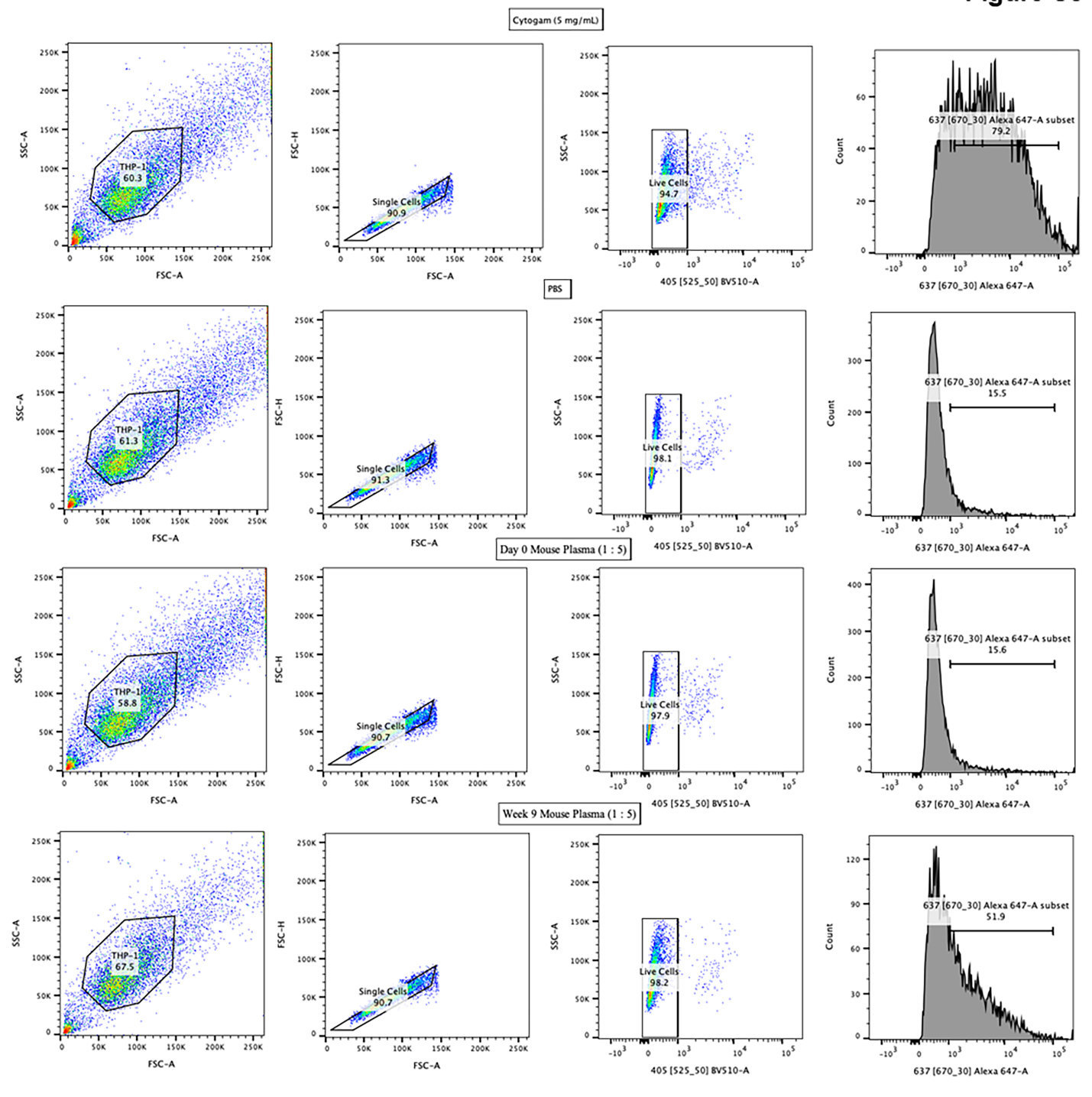
Figure S2: Flow cytometry gating strategy for measuring antibody dependent cellular phagocytosis (ADCP) activity.** An optimized amount of CMV virions were conjugated to AF647 NHS ester prior to incubation with diluted sera samples, followed by incubation with THP-1 cells. Cells were stained with Aqua Live/Dead stain, fixed with 10% formalin, and washed prior to acquisition on the flow cytometer (Fortessa; BD) using the HTS. The percentage of AF647+ cells was reported for each sample based on the live, singlet population, and the average %AF647 of the PBS wells was subtracted from the %AF647 values of controls and study samples for background correction. The average + 3SD of baseline (Week 0) samples was used as the positivity cutoff. The figure shows gating strategy for a positive control Cytogam, PBS, and mouse plasma at Day 0 (background) and Week 9.

**
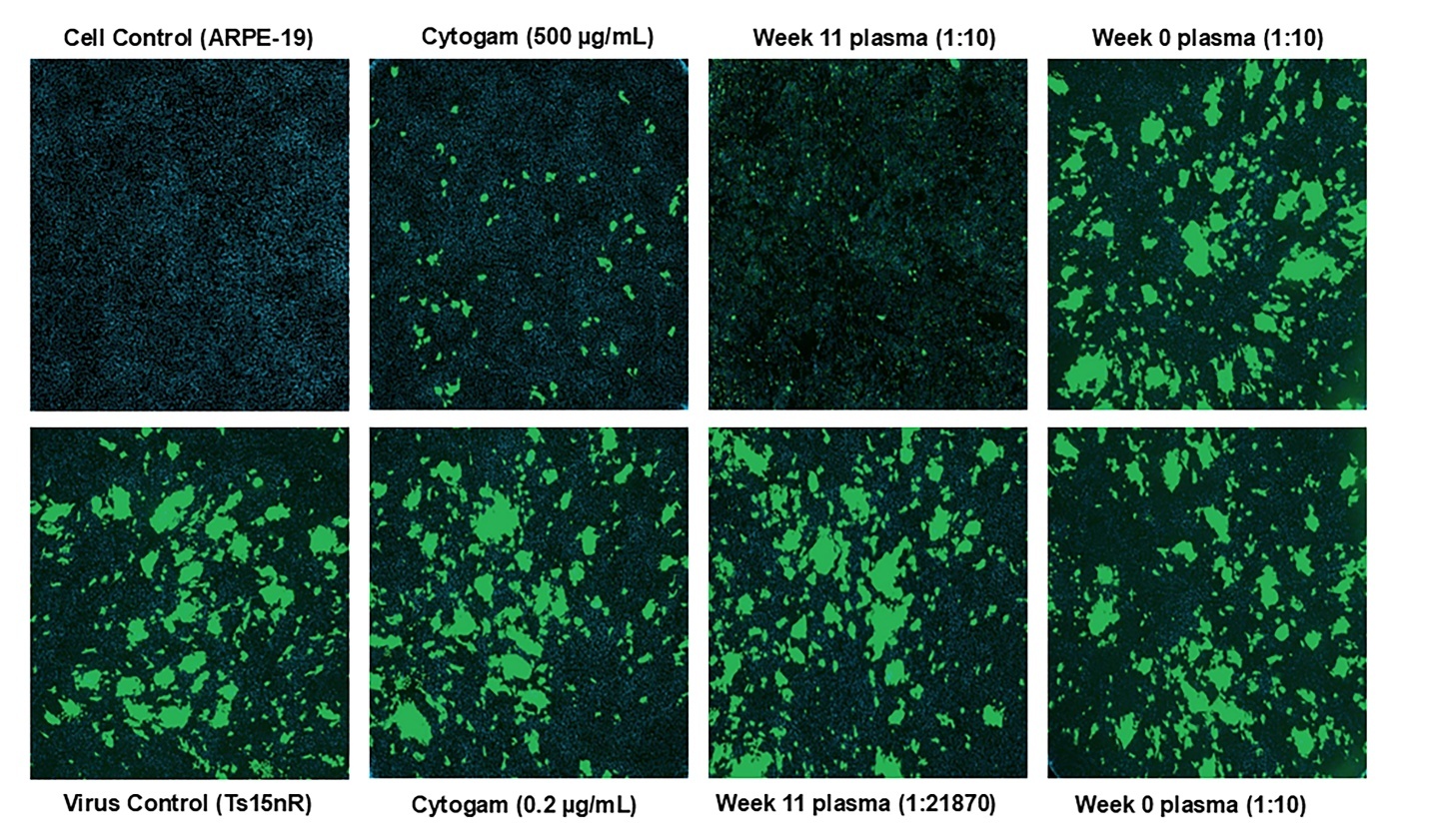
FigureS3: Representative images depicting reduction in cell-associated virus spread of Ts15nR.** ARPE-19 cells were infected with Ts15nR HCMV (MOI=0.05) overnight in 384-well plates. Virus was removed after 24 hours and replaced with media-containing either plasma dilutions (1:10 starting dilution),Cytogam, or media alone. Cells were stained for IE-1 expression and DAPI 12 days post infection and images were acquired and analyzed using the ImageExpress Pico Automated Cell Imaging Platform (Molecular Devices). Images show cell control (ARPE-19), Virus control (Ts15nR), and cell-associated virus spread for Cytogam at two concentrations, as well as Mouse plasma at Week 0 and Week 11.

**
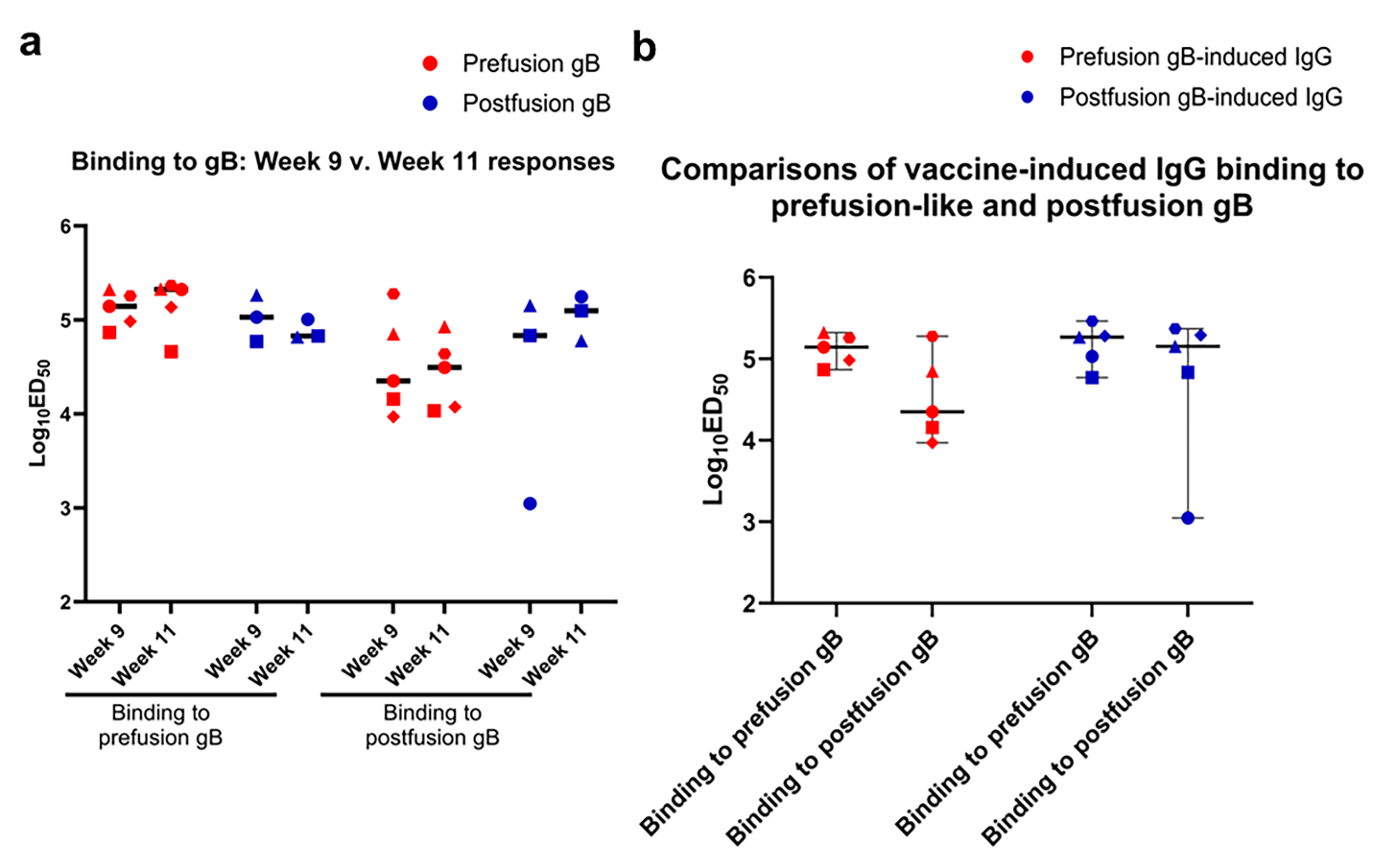
Figure S4: Vaccine-induced IgG binding to soluble and cell-associated gB** (a) Prefusion and postfusion gB immunization-induced IgG binding to soluble antigens prefusion and postfusion gB was assessed using an ELISA and reported as Log_10_ED_50_. (b) Plasma IgG binding to Prefusion-like gB-C7, and Postfusion gB was compared at weeks 9 and 11 using an ELISA and reported as Log_10_ED_50._ Symbols denote individual mice. All comparisons were done using Wilcoxin ranked sum test in Graphpad Prism.

**
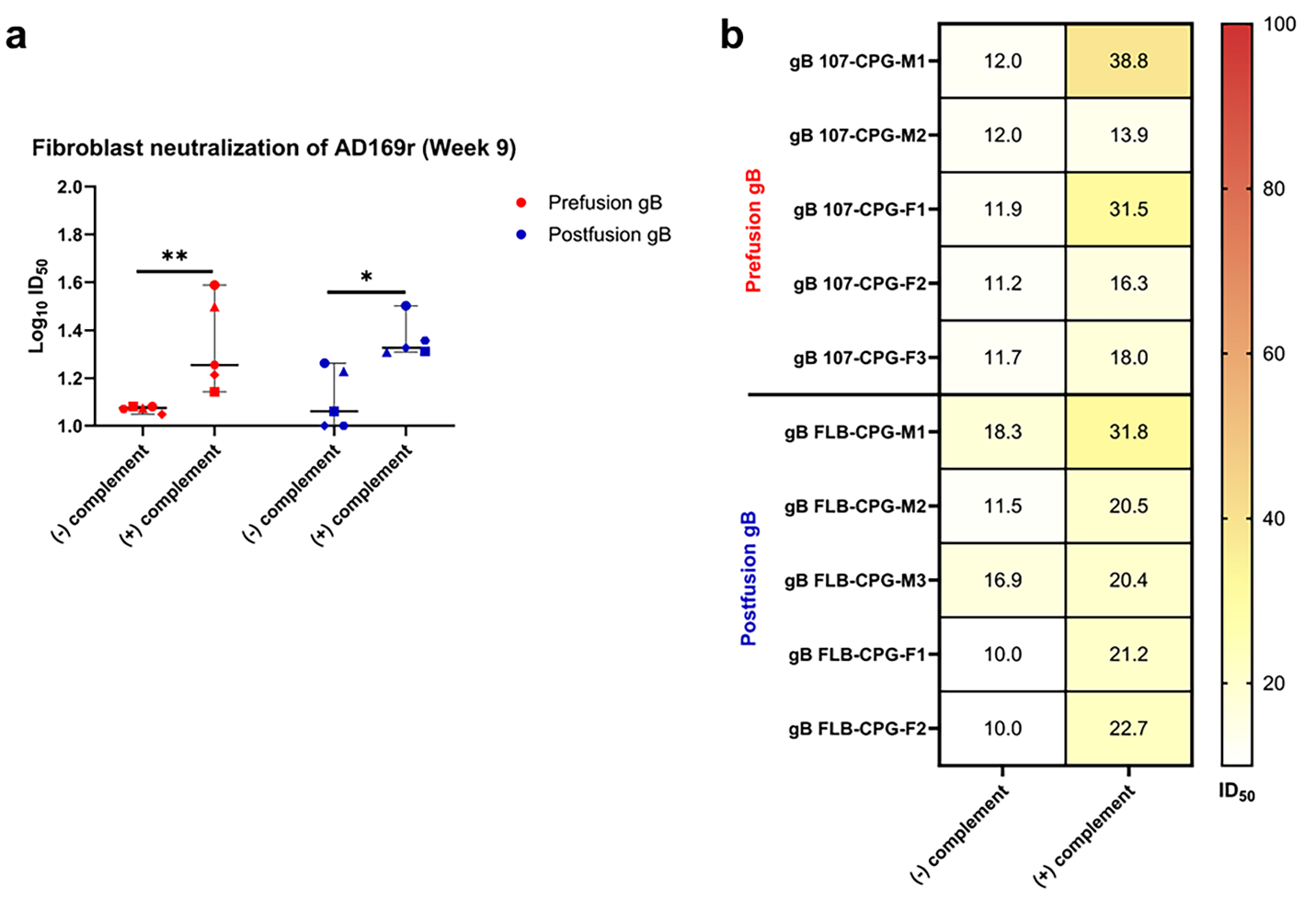
Figure S5: Complement enhances fibroblast neutralization of AD169r elicited by prefusion-like and postfusion gB vaccination.** (a) Fibroblast neutralization of AD169r was assessed without and with the addition of rabbit complement (1:8) in both prefusion and postfusion gB vaccine groups and reported as Log_10_ID_50_ . (b) Heat map representing the ID_50_ of fibroblast neutralization of AD169r assessed in plasma of individual mice without (-) and with (+) complement. *p<0.05, **p<0.01. All comparisons were done using Wilcoxin ranked sum test in R.

**
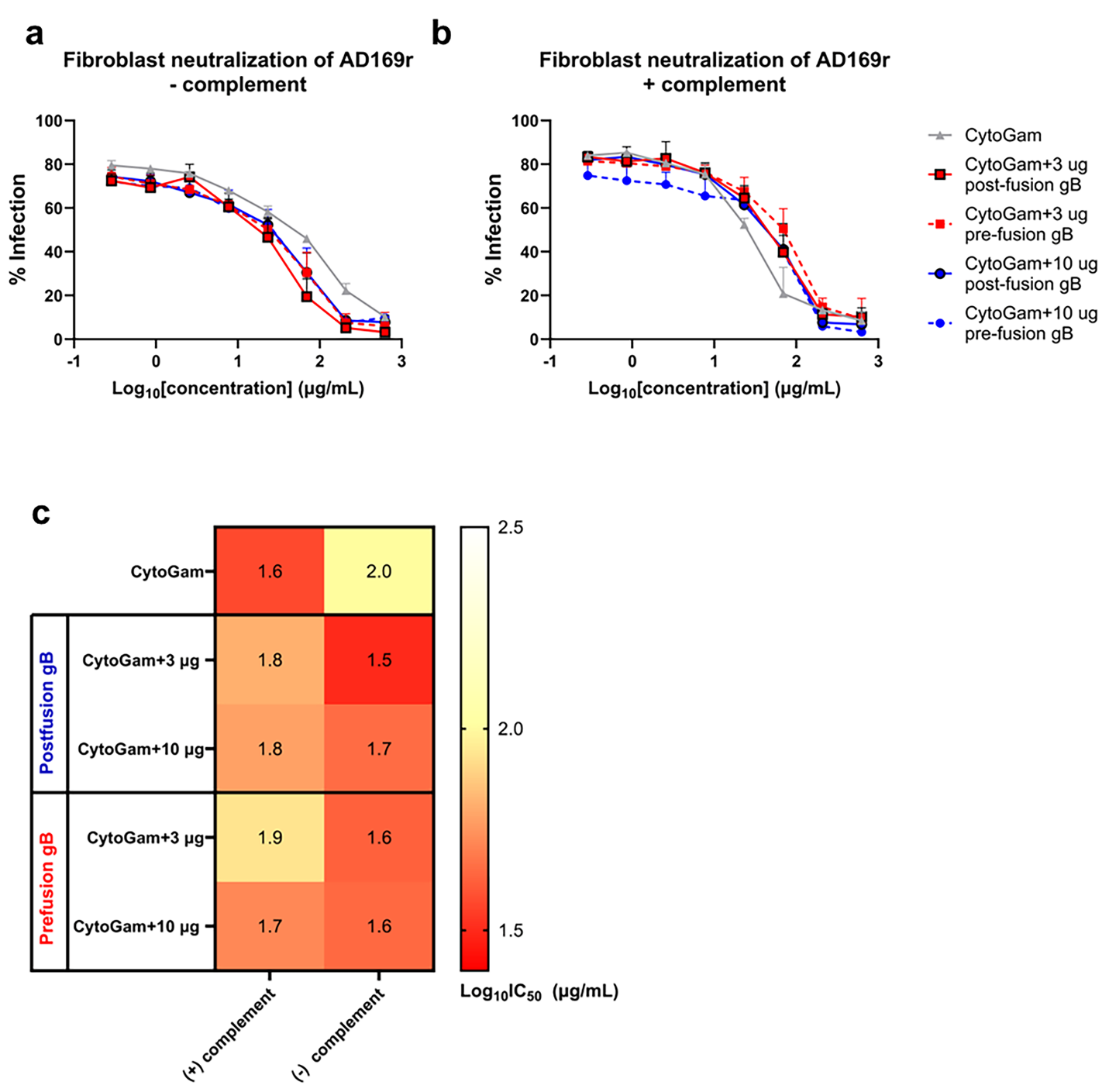
Figure S6: Prefusion or postfusion gB do not block neutralizing activity of cytogam.** Fibroblast neutralization of AD169r elicited by Cytogam was assessed alone or in the presence of 3 µg (dotted line) or 10 µg (solid line) prefusion or postfusion gB (a) without complement and (b) with the addition of rabbit complement. (c) Heat map depicting the Log_10_IC_50_ of Cytogam-induced neutralization of AD169r when blocked with prefusion or postfusion gB antigens with and without complement is shown.

**
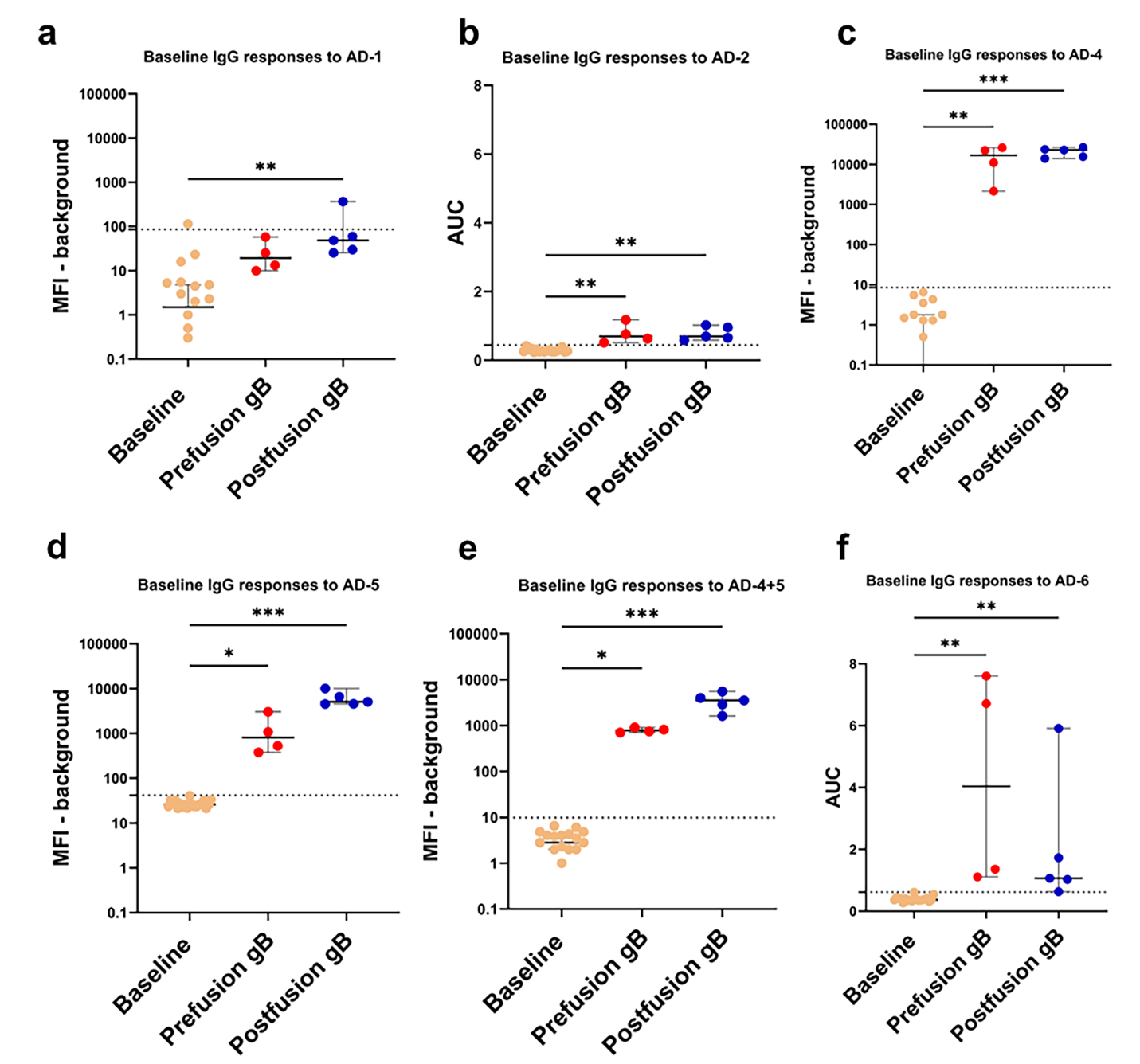
Figure S7: Baseline mouse IgG binding to gB antigenic domains.** Baseline IgG binding to (a) AD-1 (b) AD-2 (c) AD-4 (d) AD-5 (e) AD-4+5, and (f) AD-6 was assessed in unpaired mouse plasma. Binding to AD-1, AD-4, AD-4+5, and AD-5 was measured using BAMA and reported as background subtracted MFI. Binding to AD-2 and AD-6 was measured using ELISA and reported as AUC. Baseline samples were used to calculate the positivity cut-off for each antigen, represented by dotted lines on the graph as average + 3 SD of baseline IgG binding. *p<0.05, **p<0.01, ***p<0.001, ns = non-significant. All comparisons were done using one-way ANOVA in Graphpad Prism.

**
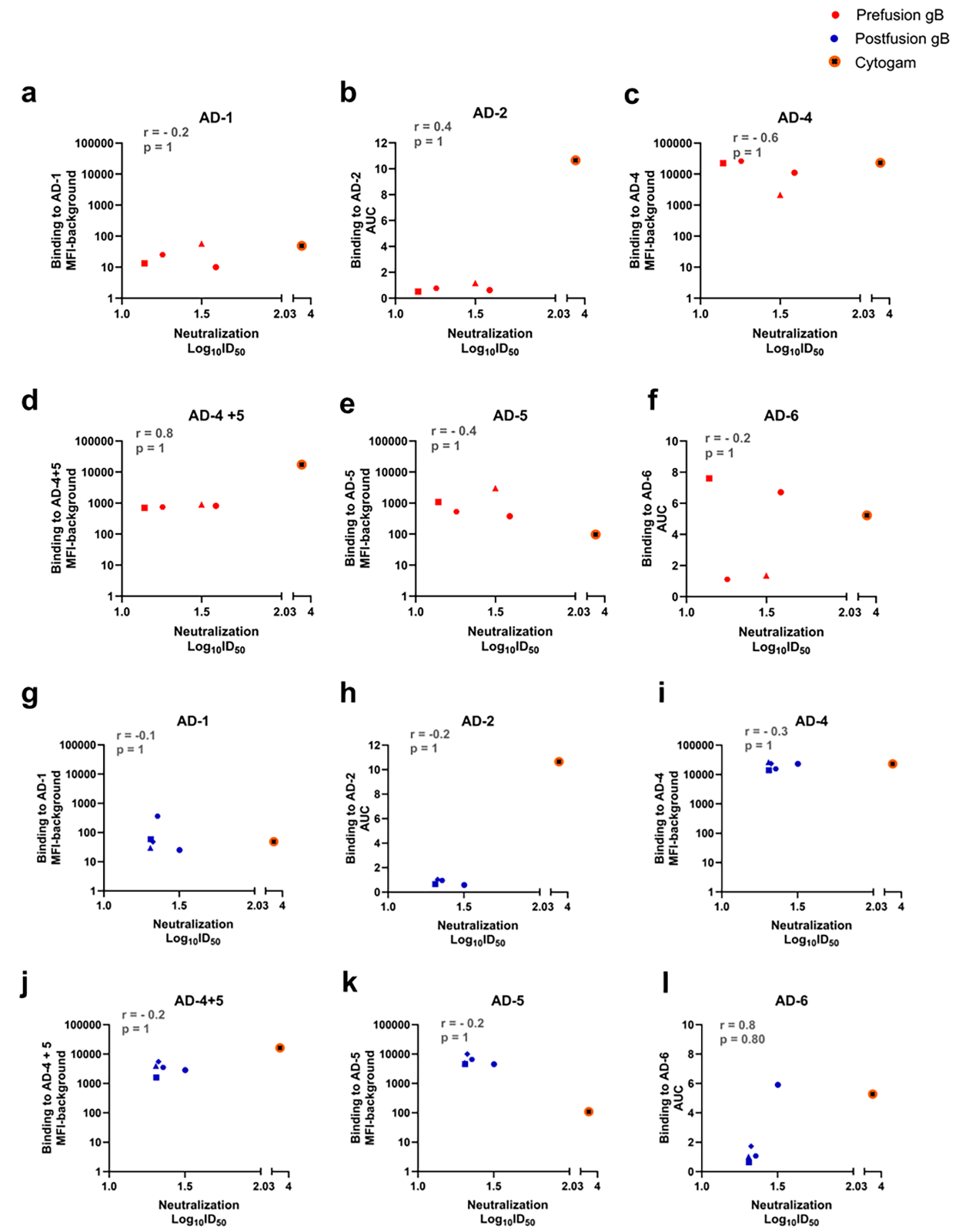
**

**Figure S8: Correlation between IgG binding to gB antigenic domains and fibroblast neutralization of AD169r with complement elicited by prefusion-like gB-C7 and postfusion gB vaccination** Correlation between prefusion-like gB induced IgG binding to (A) AD-1, (B) AD-2, (C) AD-4, (D) AD-4+5, (E) AD-5, and (F) AD-6, postfusion gB induced IgG binding to to (G) AD-1, (H) AD-2, (I) AD-4, (J) AD-4+5, (K) AD-5, and (L) AD-6, and fibroblast neutralization of AD169r with the addition of complement induced by Prefusion gB immunization was assessed using Spearman’s non-parametric correlation test in R. Insets report Spearman’s r and p values. Cytogam is plotted as a reference but was not used in correlation analysis.

**
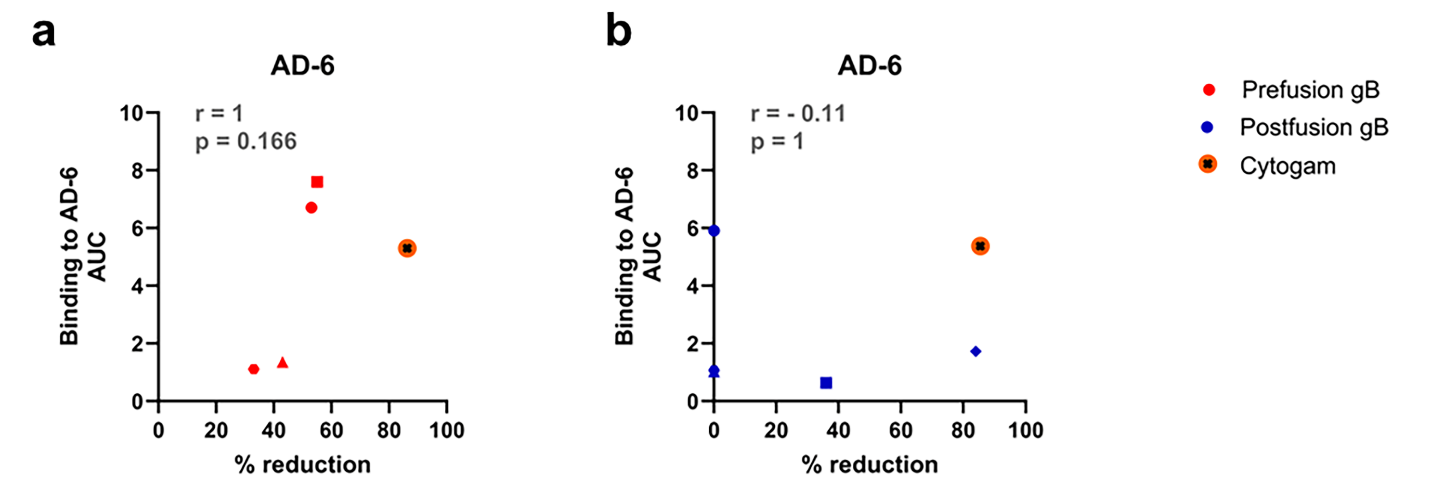
Figure S9: Correlation between IgG binding to AD-6 and reduction of cell-associated virus spread of Ts15nr** Correlation between IgG binding to AD-6 (AUC) and %reduction of cell-associated virus spread of Ts15nr induced by (a) Prefusion gB and, (b) Postfusion gB immunization was assessed using Spearman’s non-parametric correlation test in R. Insets report Spearman’s r and p values. Cytogam is plotted as a reference but was not used in correlation analysis.

| Humoral Response | Prefusion gB immunized mice  (n = 3-5) | Postfusion gB immunized mice  (n = 3-5) | p-value | FDR-adjusted p-value |
| --- | --- | --- | --- | --- |
|  | Median (Range) | Median (Range) |  |  |
| **IgG binding** |  |  |  |  |
| *Soluble gB-specific (Log_10_ED_50_)* |  |  |  |  |
| Binding to prefusion gB |  |  |  |  |
| Week 9 | 5.146 (4.867 , 5.324) | 5.267 (4.772, 5.466) | 0.690 | 0.841 |
| Week 11 | 5.326 (4.662 , 5.368) | 4.831 (4.818 , 5.007) | 0.25 |  |
| Binding to postfusion gB |  |  |  |  |
| Week 9 | 4.352 (3.971 , 5.277) | 5.155 (3.048 , 5.373) | 0.421 | 0.841 |
| Week 11 | 4.496 (4.033 , 4.640) | 5.098 (4.782 , 5.247) | 0.071 |  |
| *Cell-associated gB (%binding)* |  |  |  |  |
| Towne | 44.38 (23.15 , 47.75) | 56.65 (50.85 , 59.10) | 0.029 | 0.056 |
| **Neutralization (Log_10_ID_50_)** |  |  |  |  |
| AD169r (fibroblast) – C | 1.075 (1.048 , 1.081) | 1.060 (1.000, 1.262) | 0.834 | 0.834 |
| AD169r (fibroblast) + C | 1.255 (1.142 , 1.589) | 1.326 (1.309 , 1.502) | 0.548 | 0.626 |
| Towne (fibroblast) + C | 1.000 (1.000 , 1.017) | 1.000 (1.000 , 1.000) | 0.617 | 0.617 |
| AD169r (epithelial) + C | 1.000 (1.000 , 1.000) | 1.062 (1.000 , 1.092) | 0.197 | 0.386 |
| TB40e (epithelial) + C | 1.000 (1.000 , 1.000) | 1.000 (1.000 , 1.000) | 0.617 | 0.617 |
| **gB AD mapping (MFI)** |  |  |  |  |
| AD-1 | 19.3 (10.0 , 57.8) | 48.5 (25.3 , 365) | 0.140 | 0.201 |
| AD-2 site 1 (AUC) | 0.693 (0.513 , 1.177) | 0.693 (0.586 , 1.025) | 0.905 | 0.905 |
| AD-4 | 16717.9 (2151.3 , 26265.0) | 23049 (14056.5 , 26453.0) | 0.413 | 0.561 |
| AD-5 | 805. 1 (378.3 , 3041.5) | 5096.5 (4531.0 , 10073.0) | 0.016 | 0.063 |
| AD-4&5 | 779.5 (702.5 , 906.8) | 3534.8 (1611.3 , 5524.0) | 0.016 | 0.063 |
| AD-6 (AUC) | 4.036 (1.109 , 7.607) | 1.069 (0.633 , 5.910) | 0.190 | 0.619 |
| **Non-neutralizing antibody functions** |  |  |  |  |
| ADCP (% Phagocytosis): AD169r | 14.075 (13 , 18.55) | 21.55 (15.75 , 23) | 0.032 | 0.032 |
| ADCP (% Phagocytosis) : Towne | 30.2 (13.9 , 35.5) | 30.0 (25.2 , 31.4) | 0.92 | 0.92 |
| % reduction in cell-associated spread | 53 (33, 58) | 0 (0, 84) | 0.205 | 0.205 |
|  |  |  |  |  |

**Supplementary Table 1: Summary of plasma humoral responses assessed in mice immunized with prefusion-like gB or postfusion gB**

MFI: Median fluorescence intensity, FDR: False discovery rate, C: Complement (rabbit)
